# Characteristic of the Ascorbate Oxidase Gene Family in *Beta vulgaris* and Analysis of the Role of AAO in Response to Salinity and Drought in Beet

**DOI:** 10.3390/ijms232112773

**Published:** 2022-10-23

**Authors:** Monika Skorupa, Joanna Szczepanek, Seher Yolcu, Justyna Mazur, Andrzej Tretyn, Jarosław Tyburski

**Affiliations:** 1 Faculty of Biological and Veterinary Sciences, Nicolaus Copernicus University, 87-100 Torun, Poland; 2 Centre for Modern Interdisciplinary Technologies, Nicolaus Copernicus University, 87-100 Torun, Poland; 3 Faculty of Engineering and Natural Sciences, Sabanci University, Istanbul 34956, Turkey

**Keywords:** ascorbate oxidase, *Beta vulgaris*, *Beta maritima*, salt stress, salt shock, drought stress, drought shock, DNA methylation

## Abstract

Ascorbate oxidase, which is known to play a key role in regulating the redox state in the apoplast, cell wall metabolism, cell expansion and abiotic stress response in plants, oxidizes apo-plastic ascorbic acid (AA) to dehydroascorbic acid (DHA). However, there is little information about the AAO genes and their functions in beets under abiotic stress. The term salt or drought stress refers to the treatment of plants with slow and gradual salinity/drought. Contrastingly, salt shock consists of exposing plants to high salt levels instantaneously and drought shock occurs under fast drought progression. In the present work, we have subjected plants to salinity or drought treatments to elicit either stress or shock and carried out a genome-wide analysis of ascorbate oxidase (AAO) genes in sugar beet *(B. vulgaris* cv. Huzar) and its halophytic ancestor *(B. maritima)*. Here, conserved domain analyses showed the existence of twelve *Bv*AAO gene family members in the genome of sugar beet. The *Bv*AAO_1–12 genes are located on chromosomes 4, 5, 6, 8 and 9. The phylogenetic tree exhibited the close relationships between *Bv*AAO_1-12 and AAO genes of *Spinacia oleracea* and *Chenopodium quinoa*. In both beet genotypes, downregulation of AAO gene expression with the duration of salt stress or drought treatment was observed. This correlated with a decrease in AAO enzyme activity under defined experimental setup. Under salinity, the key downregulated gene was *Bv*AAO_10 in *Beta maritima* and under drought the *Bv*AAO_3 gene in both beets. This phenomenon may be involved in determining the high tolerance of beet to salinity and drought.

## 1. Introduction

Ascorbate oxidase (AAO) is an enzyme belonging to the blue copper oxidase family and regulates the redox state of the ascorbate pool in apoplasts [1,2]. AAOs are localized in the cell wall [3] and participate in the oxidation of apo-plastic ascorbate (AA) to mono-dehydroascorbic acid (MDHA), which is converted to dehydroascorbic acid (DHA), and then AA is generated by DHARs in the cytoplasm. The AA exchange between apoplast and cytoplasm is involved in the maintenance of redox homeostasis [1,4]. Although the AAO may be considered disadvantageous due to its role in the consumption of ascorbate levels [5], this enzyme has been found to play an important role in cell growth [5], cell wall metabolism [6] and stress responses [3,7,8]. Under abiotic stress conditions, AA is quickly oxidized to DHA and the redox state in the apoplast and cytoplasm is impaired, regulating in stress responses through ABA synthesis and H_2_O_2_ accumulation [4,9]. Contrary to ascorbate peroxidases (APXs) that use AA to scavenge ROS and decrease oxidative stress, AAOs induce ROS production/synthesis in the apoplast. Thus, overexpression of the AAO gene leads to decreased abiotic stress tolerance through increased ROS levels and disrupts stress signal transduction from the apoplast to the cytoplasm [7,10].

The regulation of signaling pathways by AAOs has been previously found in cotton [11]. Pan et al. (2019) identified eight AAO genes from *Gossypium hirsutum (GhAO)*. Bioinformatic analyses revealed that GhAOs are involved in plant development, light response and leaf senescence [11]. Contrary to *G. hirsutum*, sugar beet AAO genes have not been previously identified and characterized. There is only one study showing the effects of AAOs on sugar beet metabolism. Singh et al. (2020) reported that the AAO-sprayed sugar beets displayed a systemic resistance against beet cyst nematode, *Heterodera schachtii*, resulting from the induction of hormonal defense pathways [8]. After infection with this nematode, sugar beet roots increased the levels of some hormones such as jasmonic acid (JA), salicylic acid (SA) and ethylene (ET). Pignocchi et al. (2006) showed that the redox state of AA in the leaf apoplast affects antioxidative enzyme activities, responses of plant growth to hormones and MAP kinases and expression of genes such as catalase, glycolate oxidase and photorespiration [3]. The expression of the AAO gene is upregulated by auxin in tobacco, which is linked with the stimulation of plant growth [2].

As economically important edible crops, sugar beets (*Beta vulgaris* L. ssp. *vulgaris*) are used for the production of refined table sugar, food additives, bioethanol and biofertilizers [12,13,14,15]. The beets originated from *Beta vulgaris* ssp. *maritima* or *Beta maritima* (sea beet or wild beet), which is highly salt-tolerant during germination and seedling stages compared to other beet cultivars and is distributed across the Mediterranean Sea and European North Atlantic Ocean [16,17,18]. Sugar beets have lower genetic diversity as compared to *B. maritima* [18,19] and therefore wild beet is a considerable resource for stress-related studies and crop improvement [20]. Although sugar beet and wild beet varieties have common stress response mechanisms, such as maintenance of leaf turgor, reduction of stomatal conductance and transpiration, accumulation of Na^+^ and Cl^-^ in leaves and an increment in proline and sucrose concentrations [21,22], they exhibit different tolerance to salt and drought conditions [16,18]. In general, wild species tend to be more tolerant to stressful conditions. This was also confirmed experimentally for the wild beet (*Beta maritima*) versus cultivated beet species [23]. Moreover, we have confirmed the higher stress tolerance of *Beta maritima* compared to *Beta vulgaris* cv. Huzar in our previous experiments [17,24,25]. Wild beet populations survive in salt marshes and seashore cliffs and hence display variations in their phenotypes [18]. They show salt tolerance traits including a higher relative growth rate (RGR), high succulence index, higher volume of the palisade and spongy parenchyma cells, smaller leaf area, more leaves, efficient osmotic adjustment and higher antioxidative enzyme activities as compared to cultivated beets [26,27,28,29,30]. Moreover, the wild beets adapt to low water potential, high levels of Na^+^ and Cl^-^ and they can regulate leaf temperature in response to stress [31]. In a previous study, some morphological parameters, such as fresh biomass, total seedling length and hypocotyl/root/cotyledon lengths under salinity were found to show ecotype-specific variations in three wild beet ecotypes and a sugar beet variety [16]. Under drought stress, *B. maritima* displayed a better performance than sugar beets with respect to specific leaf weight, which is associated with leaf thickness [27,32]. In the present work, we have used the wild halophytic ancestor of beet *(B. maritima)* and sugar beet *(B. vulgaris)* cv. Huzar, which is a moderately salt-tolerant species and has been reported to show differential responses to salt stress at biochemical and molecular levels compared to *B. maritima* [17,24].

Salinity and drought stress are major constraints that decrease sugar beet growth and production [23,33]. Salt or drought stress results from treating plants with relatively slow and gradual increments in salinity/drought [34,35]. In contrast, salt shock occurs when, plants are exposed instantaneously to high salt. Consistently, a rapid desiccation imposed to plants results in a drought shock [35]. Although sugar beet is a highly salt- and drought-resistant crop plant [17,36], salt and drought stress cause yield loss and reduction in growth [37,38,39,40]. Hence, understanding the abiotic stress response mechanisms at the molecular level for this crop is required. Recently, it has been demonstrated that epigenetic marks, including DNA methylation and histone modifications, play a crucial role in the regulation of gene expression in response to abiotic stress [41,42]. DNA cytosine methylation is a transcriptional switch, controlling the activity of effector genes [43]. It is correlated with the repression of gene expression. DNA methylation was altered in different regions of stress-responsive genes in *Arabidopsis* [44], soybean [45], rice [46] and tobacco [47], etc. In a recent study, salt stress brought about the alterations in DNA methylation levels of gene promoters and first exons of salt-inducible genes in sugar beet and sea beet [42]. DNA methylation levels were reduced under salt stress and shock in *B. vulgaris*, but increased in response to salt stress in *B. maritima* [42]. These findings suggest the involvement of DNA methylation in salt-tolerance and gene expression in beets under salt stress. However, there is little information about epigenetic regulation of abiotic stress response in sugar beet and *Beta maritima* [23,42,48]. Hence, in this paper, we have detected DNA methylation patterns along with AAO transcript levels under salt/drought stress and shock treatments.

The aim of the study was to characterize AAO genes in *Beta vulgaris* and to analyze the probable role of AAO in response to salinity and drought in *Beta vulgaris* cv. Huzar and *Beta maritima*. Firstly, AAO genes were identified in *B. vulgaris* and their chromosomal positions and conserved domain protein family were investigated. A phylogenetic tree and phylogram were constructed using AAO amino acid sequences from *B. vulgaris, Spinacia oleracea* and *Chenopodium quinoa.* In addition, the transcript levels of AAO genes *Bv*AAO_3, *Bv*AAO_4 and *Bv*AAO_10 in leaves were determined under stress and shock. Moreover, DNA methylation patterns in CpG islands localized in promoter regions of *Bv*AAO_3, *Bv*AAO_4 and *Bv*AAO_10 genes were compared in *B. vulgaris* and *B. maritima* after 3, 7 and 11 days of stress and shock. Furthermore, the concentration of total ascorbate (ASC) and the activity of the AAO enzyme were determined. This is the first study aimed at investigating the characteristics of AAO members in *Beta vulgaris*. The research results summarized in this study might contribute to better understanding the responses of AAO genes to salt and drought stress in sugar beet and its wild ancestor, *Beta maritima*.

## 2. Results

### 2.1. Characteristics of Genes and Proteins of Beta vulgaris Ascorbate Oxidases

**Figure 1:**
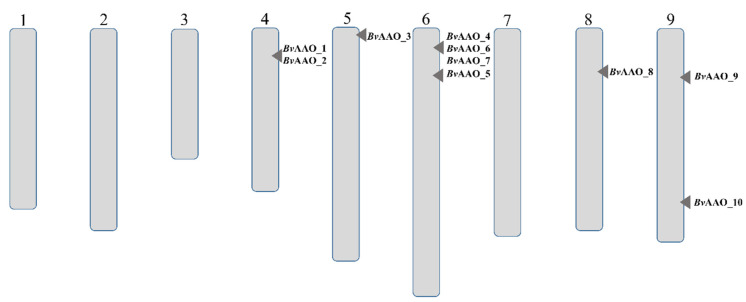
Chromosomal positions of AAO genes in *Beta vulgaris*.

Analysis of the RefBeet-1.2.2 beet reference genome deposited with GenBank allowed the identification of 12 ascorbate oxidase genes located on chromosomes 4, 5, 6, 8 and 9 (Figure 1). The two genes encoding this enzyme have not been assigned a chromosomal location. The exact characteristics of individual genes and the proteins they encode are presented in Table 1. For the purposes of this study, the genes were marked with consecutive numbers that follow the abbreviation of the enzyme name.

**Figure 2:**
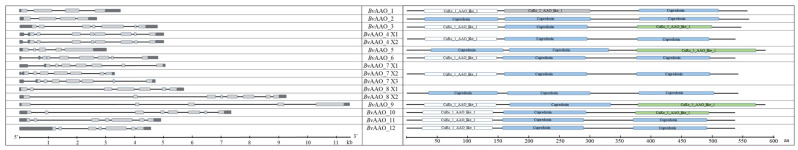
Exon−intron structure (**left** side) and conserved protein domain family (**right** side) of AAO genes in *Beta vulgaris*. UTRs−dark gray rectangles, exons−light gray rectangles, introns−black lines, the first cupredoxin domain of plant ascorbate oxidase homologs−white rectangles, the second cupredoxin domain of plant ascorbate oxidase homologs−gray rectangle, the third cupredoxin domain of plant ascorbate oxidase homologs−green rectangles, cupredoxin superfamily domain−blue rectangles.

Figure 2 shows the structure of individual beet AAO genes. They differ significantly in the number and length of exons and introns. The *Bv*AAO_8 and *Bv*AAO_9 genes are the longest. The shortest were *Bv*AAO_2 and *Bv*AAO_5. 

The proteins encoded by the AAO genes are composed of 537–588 amino acids (Table 1). Their molecular weight falls within a range of 59.68–65.9 kDa and the isoelectric point is between 6.68–9.84. According to computer analysis, the signal peptide is detected in all proteins except *Bv*AAO_9. According to the outcome of Plant-mPLoc analysis, all beet AAO proteins are located in the cell membrane. In turn, analyzes using the YLoc program indicated the cytoplasmic localization of the *Bv*AAO_2, *Bv*AAO_4, *Bv*AAO_5, *Bv*AAO_6, *Bv*AAO_8, *Bv*AAO_9 and *Bv*AAO_10 proteins; peroxisome localization in the case of *Bv*AAO_1, *Bv*AAO_3. The *Bv*AAO_7 and *Bv*AAO_11 proteins were identified by the program in the extracellular space and the *Bv*AAO_12 protein was characterized by the secretory pathway (Table 1).

Conserved protein domains of cupredoxins have been detected in beet AAO proteins. These domains contain copper centers and are involved in intermolecular electron transfer reactions. Depending on the copper-binding site, there are types I, II and III of such domains (CuRo_1_AAO_like_1; CuRo_2_AAO_like_1; CuRo_3_AAO_like_1). The domain of the cupredoxin superfamily (Cupredoxin) also has a type 1 copper center. Although the AAO proteins may have different functions, 3 cupredoxin domains were detected in each of them. The presence of the different types of domains has an equal configuration for the individual proteins (Figure 2).

### 2.2. Phylogenetic and Evolutionary Analyses of AAO Gene Family

**Figure 3:**
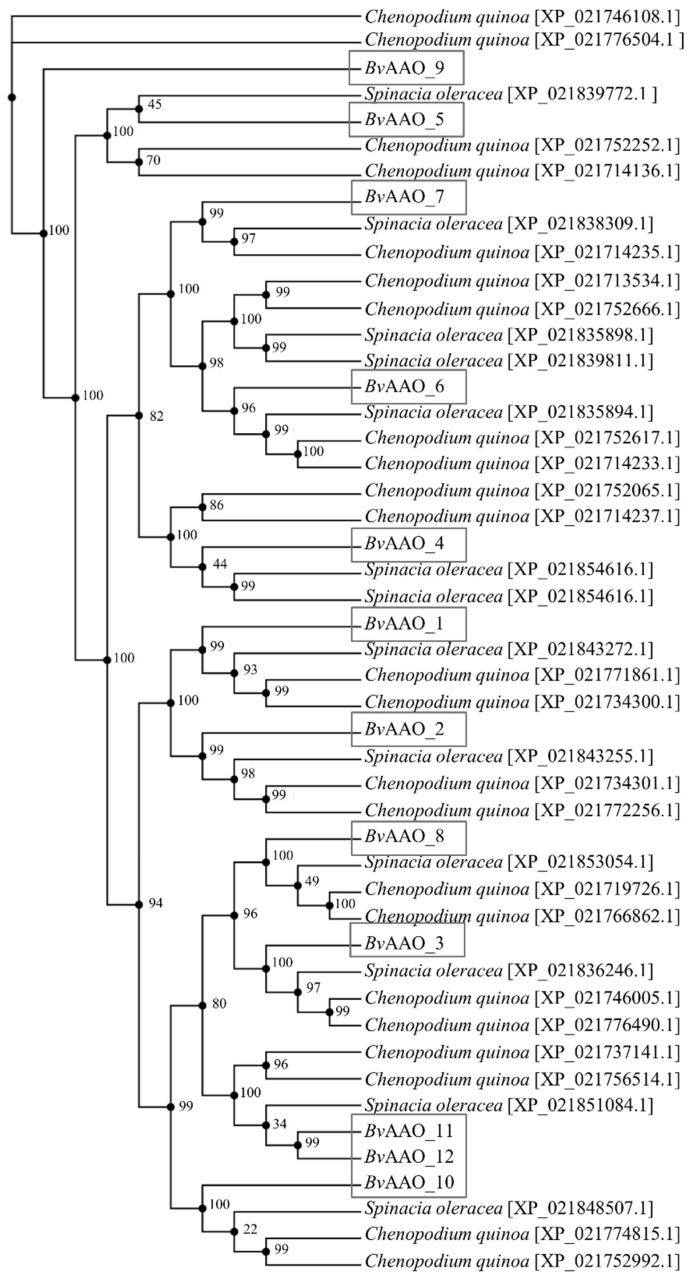
Phylogenetic analysis of AAO genes. Trees were built using amino acid sequences from *Beta vulgaris, Spinacia oleracea* and *Chenopodium quinoa.* The linear phylogram was constructed using the neighbor−joining method and bootstrapped 1000 times. Beet AAO genes are marked with gray boxes.

A phylogenetic tree was constructed based on the AAO *Beta vulgaris* protein sequences and according to the highest alignment index of the AAO protein sequences of *Spinacia oleracea* and *Chenopodium quinoa*. Based on the analysis of the reference genomes of these species, 13 spinach ascorbate oxidase sequences and 23 *Chenopodium quinoa* sequences were compared. Analyzing the evolutionary similarity of the beet AAO sequence, it can be noticed that the *Bv*AAO_9 protein is evolutionarily most distant from the rest of the proteins. In addition, no close structural similarity to any of the AAO proteins of spinach and quinoa was demonstrated. Taking into account the next phylogenetic branch, we can distinguish here known groups, i.e., *Bv*AAO_5 and the rest of the beet AAO proteins. Noticeable here also is the closer similarity of *Bv*AAO_5 to the spinach protein sequence XP_021839772.1 and then to the two quinoa sequences XP_021752252.1 and XP_021714136.1. On the subsequent branches of the tree, individual beet AAO proteins are presented, which have their counterparts among the ascorbic oxidase proteins in the two plant species analyzed (Figure 3).

### 2.3. Analysis of the AAO Beta vulgaris Gene Expression Profile under Drought and Salinity Conditions

The expression level of AAO genes was determined in salt/drought shocked/stressed leaves of *Beta vulgaris* cv. Huzar and *Beta maritima.* Untreated controls were analyzed in line with the treated plants. Since only the *Bv*AAO_3, *Bv*AAO_4 and *Bv*AAO_10 ascorbate oxidase genes were transcriptionally active in the analyzed tissue, the results of the expression analyzes are presented only for these genes.

**Figure 4:**
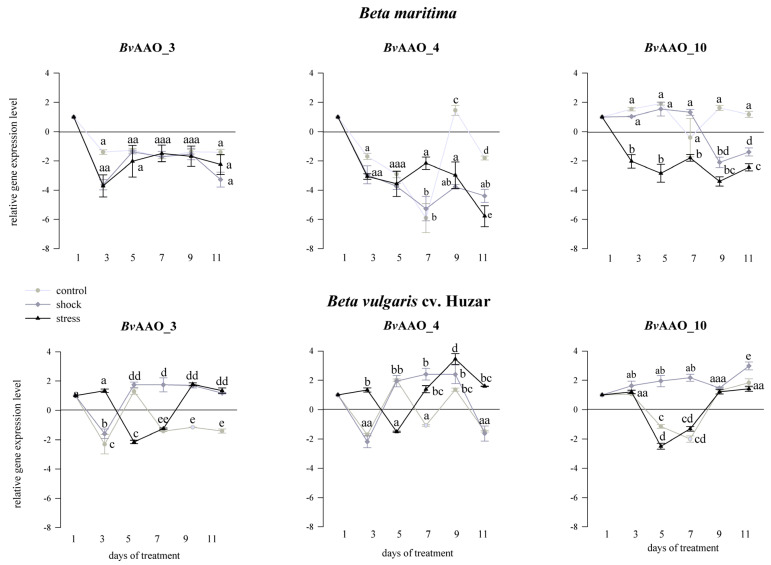
Expression profiles of AAO genes *Bv*AAO_3, *Bv*AAO_4 and *Bv*AAO_10 determined in leaves of *Beta vulgaris* ssp. *maritima* and *Beta vulgaris* cv. Huzar subjected to salt shock or stress. Control−light gray lines, salt shock−gray lines, salt stress−dark gray lines. Different letters denote significant differences at *p* < 0.01 (ANOVA followed by Tukey’s test). “Whiskers” indicate standard deviation.

When compared to the treatment starting day (“day 1”) a downregulation of *Bv*AAO_3 expression was observed, in the control, shock or salt stress treated *Beta maritima* leaves, the reduced expression level maintained over the treatment period. There were no significant differences in *Bv*AAO_3 expression levels between the control samples and those from plants treated with stress and salt shock. On the 3rd day, in the leaves of *Beta vulgaris* cv. Huzar *Bv*AAO_3 expression was observed to be downregulated in the control and salt shock treated samples relative to day 1. In turn, the expression of *Bv*AAO_3 in the samples treated with salt stress was upregulated (Figure 4). On the 5th day, the opposite effect was observed, i.e., an increase in *Bv*AAO_3 expression in control plants and those representing salt shock and a significant decrease for the salt stress variant. This downregulation persisted until the 7th day of the treatment, after which an increase in the level of expression was observed until the end of the experiment. In the controls on the 7th day, there was a decrease in the expression level of *Bv*AAO_3, which remained at a similar level on the 9th and 11th days. In the case of the samples representing salt shock, the upregulation of expression of this gene on the 5th day of treatment was maintained until the end of the experiment.

**Figure 5:**
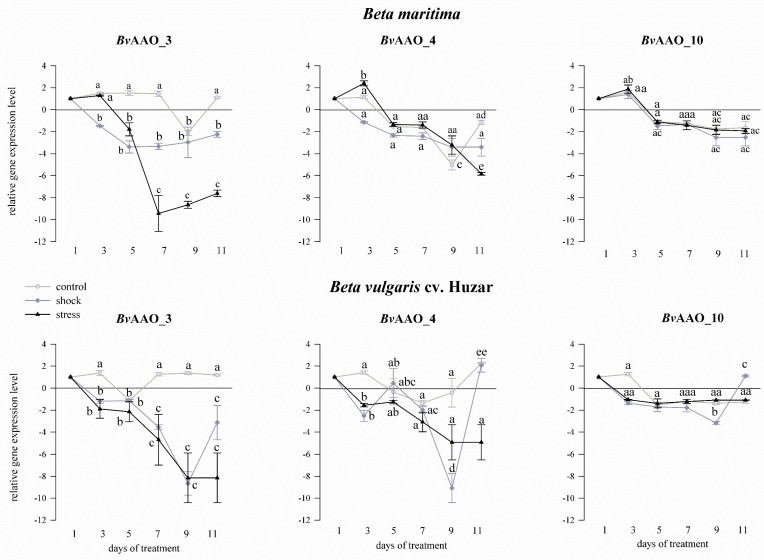
Expression profiles of AAO genes *Bv*AAO_3, *Bv*AAO_4 and *Bv*AAO_10 determined in leaves of *Beta vulgaris* ssp. *maritima* and *Beta vulgaris* cv. Huzar subjected to drought shock or stress. Control−light gray lines, drought shock−gray lines, drought stress−dark gray lines. Different letters denote significant differences at *p* < 0.01 (ANOVA followed by Tukey’s test). “Whiskers” indicate standard deviation.

When the plants were treated with drought, the expression profile of the *Bv*AAO_3 gene was different. In control samples, the transcript level in the two beet species was similar and did not change much. On the other hand, in the samples representing plants treated with shock and drought stress, a significant downregulation of *Bv*AAO_3 expression was observed, as compared to the controls, at each sampling point (Figure 5).

Analyzing the effect of salinity, the expression of the *Bv*AAO_4 gene in *Beta maritima* leaves was downregulated, relative to “day 1”, at all time-points of the experiment for the samples representing both the salt shock and salt stress treatment. The lowest transcript level was observed on the 11th day of the treatment. In the samples coming from control plants, the effect was similar except on day 9 when a slight increase in expression was observed. In leaves of *Beta vulgaris* cv. Huzar *Bv*AAO_4 expression level varied depending on the treatment day and variant of the experiment. Relative to “day 1”, downregulation of this gene expression was observed on the 3rd, 7th and 11th day of the experiment, while on the 5th and 9th day a significant upregulation was observed in the control leaves. A similar profile of changes was observed under the salt shock treatment, except on the 7th day, in which the increase in the level of the *Bv*AAO_4 transcript was maintained. In the case of samples representing salt stress on the 5th day, a decrease in the expression of this gene was observed, while on the remaining days of treatment a significant increase occurred (Figure 4).

Treating *Beta maritima* plants with drought, except for the stress variant on the 3rd day, brought about a decrease in the expression of the *Bv*AAO_4 gene, observed in all experimental variants and on all treatment days. In leaves of *Beta vulgaris* cv. Huzar, the decrease in *Bv*AAO_4 expression level was also observed in the stressed plants. The samples representing control plants showed unchanging transcript levels, except on the 11th day, when a slight increase was detected. Under drought shock, *Bv*AAO_4 levels were similar to that observed in the stress variant on the 3rd, 5th and 7th days, but turned on the 11th day to the control level (Figure 5).

Taking drought into account, the expression level of *Bv*AAO_10 in *Beta maritima* did not change significantly, as compared to “day 1”, in the samples representing control plants and those treated with salt shock up to the 7th day of the experiment. On the 9th and 11th days of treatment, a significant decrease in salt-shocked plants was observed. In contrast to the remaining variants, significant decreases in the level of the *Bv*AAO_10 transcript were observed in plants treated with salt stress on each measurement day. In *B. vulgaris* cv. Huzar expression level of *Bv*AAO_10 did not change compared to the control in the case of salt shock and on the 3rd, 9th and 11th days in the rest of the experimental variants. Significant downregulation of *Bv*AAO_10 expression occurred on the 5th and 7th day of the experiment in control plants and plants treated with salt stress (Figure 4). In the drought testing experiment, the expression level of *Bv*AAO_10 remained at a similar level compared to the control in all experimental variants in both beet genotypes (Figure 5).

### 2.4. Analysis of the Methylation Level of the CpG Islands of the Promoter Regions of the AAO Beta vulgaris Genes under Drought and Salinity Conditions

**Figure 6:**
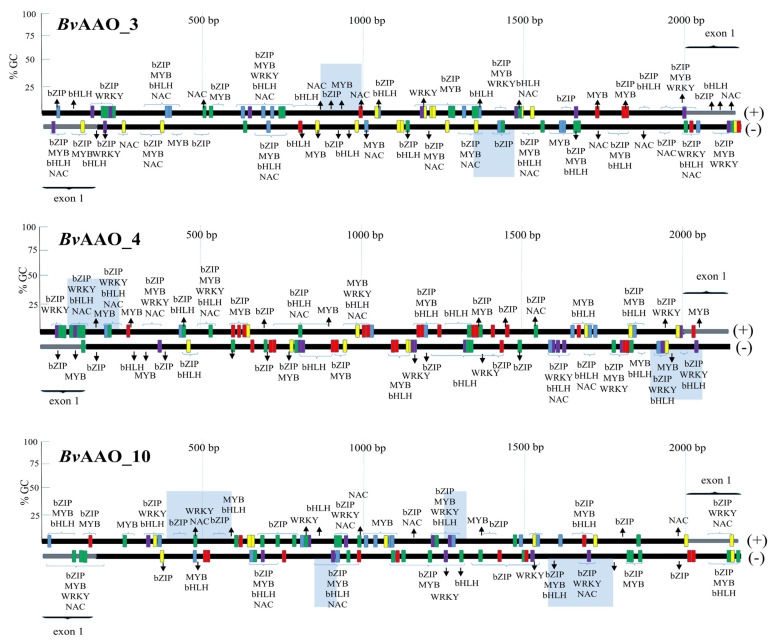
Structure and motif composition of the promoter regions of AAO genes *Bv*AAO_3, *Bv*AAO_4 and *Bv*AAO_10. The analyses were performed on sugar beet sequences derived from the RefBeet-1.2.2 reference genome. CpG islands−light blue squares, selected cis−regulatory sequence elements (TATABOX−red squares, CAATBOX−green squares, WRKY−purple squares, MYB−yellow squares, GATABOX−blue squares) and potential places recognized by specific stress−related transcription factors (bZIP, MYB, WRKY, bHLH, NAC) are shown. “+”−sense strand; “−” antisense strand.

Figure 6 shows the location of the CpG islands of the promoter regions of the *Bv*AAO_3, *Bv*AAO_4 and *Bv*AAO_10 genes. Importantly, numerous sequences containing transcription factors (TFs)-binding motifs were identified in the analyzed regions. Among them, the sites binding TFs involved in the salinity and/or drought response, i.e., representing the bZIP, MYB, WRKY, NAC and bHLH families, were present (Figure 6).

**Figure 7:**
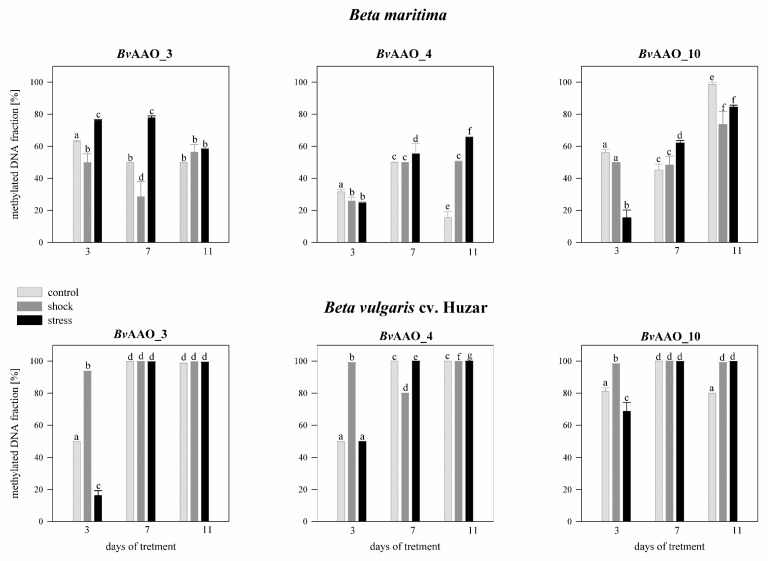
Methylated DNA fraction [%] of CpG islands in the promoter region of AAO genes *Bv*AAO_3, *Bv*AAO_4 and *Bv*AAO_10 determined in leaves of *Beta vulgaris* ssp. *maritima* and *Beta vulgaris* cv. Huzar subjected to salt shock or stress. Control−light gray bars, salt shock−gray bars, salt stress−black bars. Different letters denote significant differences at *p* < 0.01 (ANOVA followed by Tukey’s test). “Whiskers” indicate standard deviation.

Analyzing the effect of salinity on the methylation level of the *Bv*AAO_3 gene CpG island in *Beta maritima* on day 3 of treatment, it was revealed that in the control plants methylation level was above 63%, whereas on the 7th and 11th days the methylation level decreased to about 50%. In the case of plants treated with salt shock, the methylation level of the CpG *Bv*AAO_3 island was about 49%, on the 7th treatment day it decreased to 28% and on the 11th day it increased to 56%. In plants treated with salt stress, on the 3rd and 7th day of the experiment the methylation level was at the level of 76–78% and then on the 11th day of treatment it decreased to the level of the other variants. In *Beta vulgaris* cv. Huzar, on the 3rd day of treatment the level of CpG *Bv*AAO_3 island methylation was 53% in the control plants, about 98% in shock-treated plants and 18% in salt-stressed. In the following days, the levels of methylation reached almost 100% in all variants of the experiment (Figure 7).

**Figure 8:**
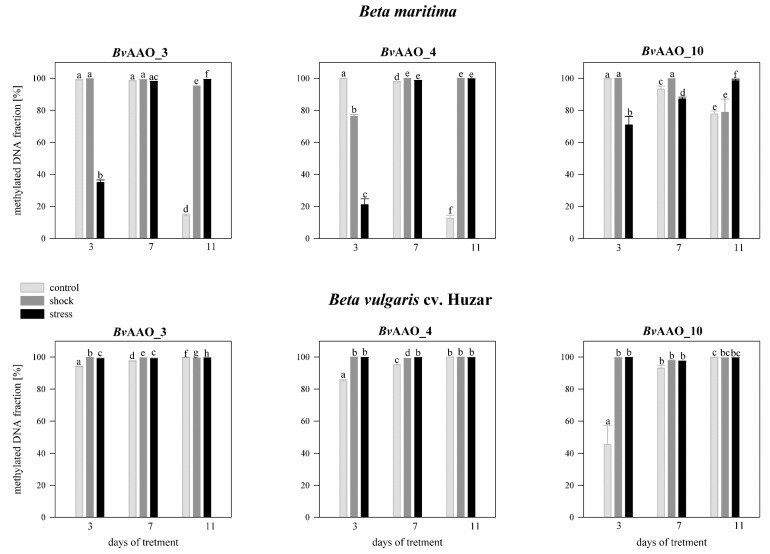
Methylated DNA fraction [%] of CpG islands in the promoter region of AAO genes *Bv*AAO_3, *Bv*AAO_4 and *Bv*AAO_10 determined in leaves of *Beta vulgaris* ssp. *maritima* and *Beta vulgaris* cv. Huzar subjected to drought shock or stress. Control−light gray bars, salt shock−gray bars, salt stress−black bars. Different letters denote significant differences at *p* < 0.01 (ANOVA followed by Tukey’s test). “whiskers” indicate standard deviation.

In drought-treated *Beta maritima* plants, the level of methylation of the CpG *Bv*AAO_3 island, measured on the 3rd day of the experiment was close to 100% in the control and drought-shock variants, while in the case of plants subjected to drought stress, it was 35%. On the 7th day of treatment, the methylation level was close to 100% in all experimental variants. It persisted on day 11 in plants treated with drought shock and stress. Contrastingly, in control plants, it decreased to the level of 14%. In *Beta vulgaris* cv. Huzar the methylation level of the CpG *Bv*AAO_3 island was close to 100% on all measurement days and in all experimental variants (Figure 8).

The methylation levels of the CpG island of *Bv*AAO_4 in *Beta maritima* on day 3 amounted to about 31% in the control plants and about 24–25% in the plants treated with salt shock or stress. On the 7th day of the experiment, the methylation level increased to about 50–55% in all experimental variants. This level was maintained on day 11 in the salt-shock treated variant. In turn, in control plants, it decreased to the level of 13%, but in those treated with salt stress it increased to the level of 65%. In *Beta vulgaris* cv. Huzar observed a high percentage of methylation of the CpG *Bv*AAO_3 island on the 7th and 11th day of treatment and on the 3rd day in the salt-shocked variant. In the remaining variants, on the 3rd day, the methylation amounted to 50% (Figure 7).

In an experiment aimed at analyzing the effect of drought on *Beta maritima*, the methylation level of the CpG island *Bv*AAO_4 in the control plants was close to 100% on the 3rd and 7th days of treatment, but it decreased to about 12% on the 11th day. In plants treated with salt shock, the methylation level on the 3rd day of the experiment was about 75% and increased to nearly 100% on the 7th and 11th days. Similarly, in the drought- stressed variant it was 21% on the 3rd day of the experiment and increased to nearly 100% in the following days of treatment. In *Beta vulgaris* cv. Huzar, the percentage of methylation of CpG *Bv*AAO_4 was very high in all experiment variants and all measurement times (Figure 8).

The level of CpG island methylation *Bv*AAO_10 in *Beta maritima,* on day 3 of treatment, was about 50–56% in the control and salt shock-treated plants and about 15% in the salt stress-treated plants. In the case of the first two variants, methylation levels decreased slightly on day 7 of the experiment and increased significantly on day 11. In plants treated with salt stress, the level of CpG island methylation in the *Bv*AAO_10 gene increased with the duration of the experiment, to 62% and 84%, respectively. In the control plants, *Beta vulgaris* cv. Huzar on the 3rd day showed an 81% methylation level of the CpG island, which increased to almost 100% on day 7 and then, on the 11th day, decreased to the level detected on the 3rd day. In plants treated with salt shock, the methylation level remained close to 100% during the course of the experiment. In the salt stress treatment variant, on the 3rd day of the experiment, the methylation level was 68% and on the 7th and 11th days it increased to almost 100% (Figure 7).

Analyzing the effect of drought revealed high methylation of CpG island in *Bv*AAO_10 gene to levels reaching near 100% in *Beta maritima* on the 3rd and 7th days of treatment in the control variant and under drought-shock. Then, a decrease was observed on the 11th day of the experiment. In plants treated with drought stress, the methylation level increased with treatment time to 70%, 87% and 98%, respectively. In *Beta vulgaris* cv. Huzar high levels of near 100% CpG island methylation *Bv*AAO_10 were observed in all variants and time points of the experiment, except for the control plants on the 3rd day (45%, Figure 8).

### 2.5. Analysis of Total Ascorbate Concentration in Beta vulgaris Leaves under Drought and Salinity Conditions

**Figure 9:**
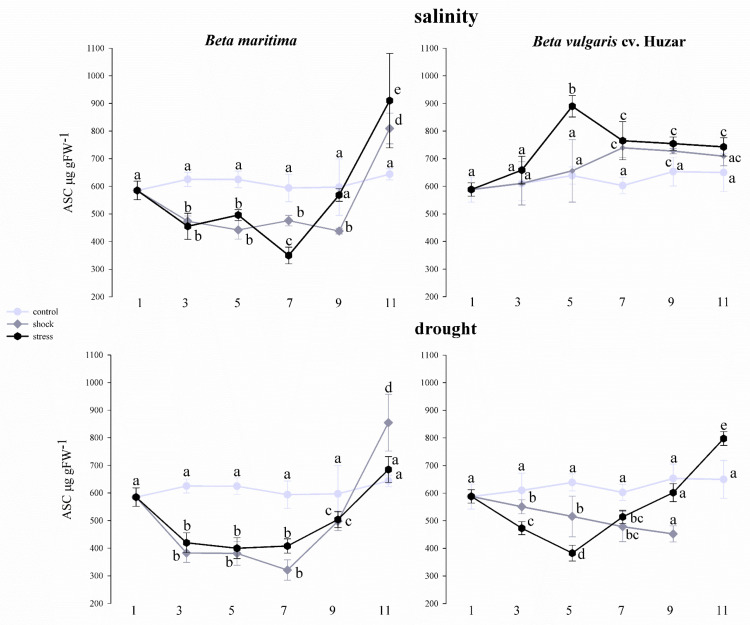
Total ascorbate concentration determined in leaves of *Beta vulgaris* ssp. *maritima* and *Beta vulgaris* cv. Huzar subjected to salt shock or stress and drought shock or stress. Control−light gray bars, salt shock−gray bars, salt stress−black bars. Different letters denote significant differences at *p* < 0.01 (ANOVA followed by Tukey’s test). “whiskers” indicate standard deviation.

Changes in ascorbate content in leaves of *Beta vulgaris* ssp. *maritima* and *Beta vulgaris* cv. Huzar were tested over a 11-day-long in plants subjected to stress-generating factors or in those maintained under control conditions, which did not involve exposing plants to stress factors. The total ascorbate levels, i.e., the sum of its oxidized and reduced forms, were determined. Leaf total ascorbate concentrations were expressed as µg ASC per gram fresh weight (FW) of leaves and are presented in Figure 9.

A similar trend of changes in ascorbate content was observed in *Beta maritima* leaves treated with both salinity and drought, irrespective of the intensity of the treatment. From the 3rd to the 9th day of the experiment, a significant decrease in ASC concentration was observed, compared to the control. The exception was the salt -treated *Beta maritima* leaves on day 9, where the ASC level was similar to that in the control. On the 11th day, in the case of salt shock and salt stress, as well as drought shock, a significant increase in ASC concentration was observed (Figure 9). In *Beta vulgaris* cv. Huzar a profile of changes was different. The leaves of plants treated with salt stress on day 5 displayed a significant increase in ASC which was maintained until the end of the experiment. In leaves treated with salt shock, similar increase was observed only from the 7th day of the experiment. Changes in ASC concentration under drought conditions were comparable to those observed in *Beta maritima*. With respect of the untreated to the control, a significant decrease in ASC concentration occurred by the 9th day of treatment, except for the leaves of plants treated with salt stress. On the 11th day of the experiment, a significant increase in ASC concentration was observed in this variant, as compared to the control. The ASC concentration for the drought shock treatment variant on day 11th was not determined due to the death of the plants (Figure 9).

### 2.6. Analysis of Ascorbate Oxidase Activity in Beta vulgaris Leaves in Drought and Saline Conditions

**Figure 10:**
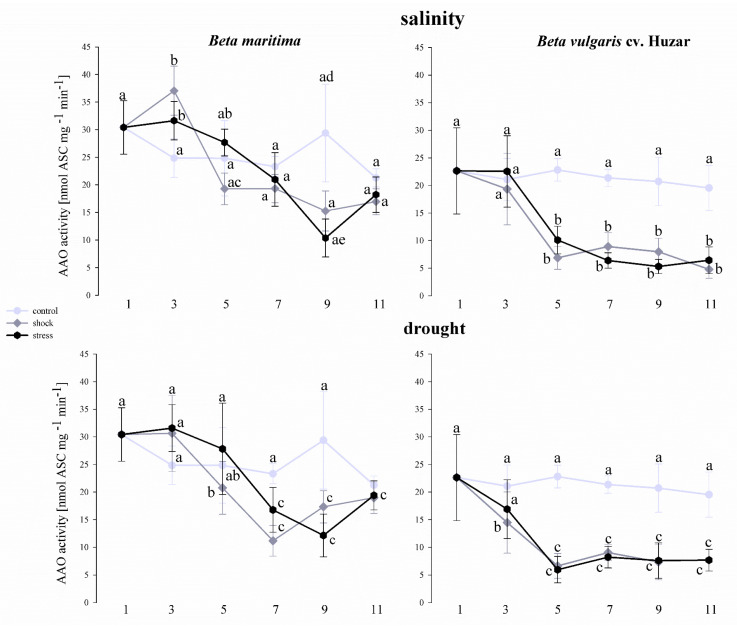
AAO activity determined in leaves of *Beta vulgaris* ssp. *maritima* and *Beta vulgaris* cv. Huzar subjected to salt shock or salt stress and drought shock or drought stress. Control−light gray bars, salt shock−gray bars, salt stress−black bars. Different letters denote significant differences at *p* < 0.01 (ANOVA followed by Tukey’s test). “Whiskers” indicate standard deviation.

The experiments also included the determination of AAO activity in two beet subspecies (Figure 10). In *Beta maritima*, an increase in enzyme activity was observed in leaves treated with both, salt shock and stress, on the 3rd day of the experiment, compared to the control. On day 5, this increase was still visible in the samples representing salt stress treatment, although the difference was statistically insignificant. Similarly, in the leaves representing the salt shock-treated leaves, there was a decrease in activity relative to the control on the 5th day of treatment, although these differences were not statistically significant. On the 7th and 11th days of the experiment, AAO activity did not differ between the variants. On day 9, a significant decrease in AAO activity was observed, relative to control, in leaves of plants treated with salt stress. The leaves treated with salt shock also showed a decrease in AAO activity, although the difference was not statistically significant when compared to untreated control. Under the drought treatment, the AAO activity was similar in all variants on the 3rd and 11th days of the experiment. On the 5th day a significant decrease in AAO activity relative to the control were observed in the samples representing drought shock-treated and on days 7th and 9th by drought shock and drought stress-treated plants. In *Beta vulgaris* cv. Huzar a significant decrease in AAO activity was observed between the 5th and 11th day of the experiment, in leaves treated with both saline and drought. Furthermore, a decrease in activity occurred in the leaves treated with drought shock on the 3rd day of treatment (Figure 10).

## 3. Discussion

AAO is a member of the polymorphic oxidase family that catalyzes redox reactions and plays an important role in many plant physiological processes. The enzyme could also have a role during stress responses modifying the ascorbic acid levels and therefore changing the cellular redox balance, which might provide the plant cell with an excellent system to sense the environment and react appropriately by contributing to the stress-related control. The expression of AAO is regulated by complex transcriptional and post-transcriptional controls, with transcript levels induced by growth promoters, e.g., auxin, jasmonates, and reduced by growth suppressors, e.g., salicylic acid [49]. On the other hand, experiments with many plants show that suppression of the AAO gene increases their stress tolerance, while overexpression has the opposite effect [50,51]. Overexpression of the AAO gene in tobacco inhibited germination and seed yield upon salt stress [51]. In another report, upregulation of cucumber AAO gene expression reduced the apo-plastic AA redox state and oxidative stress tolerance in tobacco plants, which is associated with the lower transcription of genes involved in AA recycling under oxidative stress conditions [52]. Transgenic tobacco overexpressing the AAO gene from cucumber displayed an increase in apo-plastic DHA accumulation, lower stomatal conductance and higher water content as compared to WT [4]. In contrast, suppression of the tomato AAO gene resulted in AA accumulation in fruits and enhanced drought stress tolerance by higher photosynthetic capacity [53]. Similarly, Yamamoto et al. (2005) reported that the transgenic tobacco plants that have suppressed AAO gene expression in antisense orientation showed increased germination, photosynthetic activity and seed yield and therefore salt tolerance compared to WT and sense plants under high salt concentrations [51].

This study aimed to characterize the ascorbate oxidase genes in *Beta vulgaris*, as well as to evaluate selected elements of the salinity and drought response at the epigenetic and transcriptional levels associated with AAO in the leaves of *Beta vulgaris* and its halophytic ancestor *Beta maritima*. Literature data show only two studies presenting the characteristics of ascorbate oxidase genes in other plant species [11,49]. Thus, there is no study of this enzyme in *Beta vulgaris*. The conducted analyses allowed distinguishing of 12 genes of beet ascorbate oxidase. Genomic characterization of other plant species revealed five AAO genes in *Oryza sativa*, three AAO genes in *Arabidopsis thaliana*, seven AAO genes in *Glycine max*, four AAO genes in *Zea mays*, six AAO genes in *Sorghum bicolor* and eight AAO genes in *Gossypium hirsutum* [11,49]. In each of the 12 beet AAO protein sequences, three from the cupredoxin domain were localized. This is consistent with the presented data of other studies characterizing the genes of ascorbate oxidases in other plant species [11,49]. Interestingly, individual proteins contain different configuration combinations of domain types, which can presumably be associated with different functions of individual AAO proteins and/or their different localization. Different computer programs point to different locations of the same beet AAO. The literature data is also inconsistent in this matter. Caputo et al. indicate that immunogold transmission electron microscopy analysis showed labeling of AAO in the cytosol, walls and chloroplasts of *Chaetomorpha linum* cells, while in angiosperms this enzyme is almost exclusively limited to the extracellular matrix [54]. According to other literature data, AAO activity is mainly related to the cell wall fraction. It has been suggested that it may play an important role in regulating the oxygen balance and controlling the growth of plant cells, perhaps by controlling transport processes through the plasma membrane [55]. Phylogenetic analysis shows that all beet AAO genes, except *Bv*AAO_9, have structural equivalents in the genes of *Spinacia oleracea* and *Chenopodium quinoa*. Interestingly, this is the only beet protein sequence in which the program failed to detect a signal peptide.

The conducted experiments showed that the *Bv*AAO_3, *Bv*AAO_4 and *Bv*AAO_10 genes were transcriptionally active in the leaves of two beet species. Sanmartin et al. also showed that of at least four AAO genes in *Cucumis melo*, only *Cm*AO1 and *Cm*AO4 were transcriptionally active. Moreover, researchers proved that their expression was regulated under the influence of wounding and heat stress [9]. The expression profiles of the *Bv*AAO_3, *Bv*AAO_4 and *Bv*AAO_10 genes are different under salinity and drought treatments. The effects of shock and stress in both treatments also have a different effect on the expression level of AAO genes. Moreover, the transcriptional response of these three genes differs in the leaves of *Beta vulgaris* and its halophytic ancestor *Beta maritima*.

When analyzing the effect of salinity on the level of gene expression in *Beta maritima*, a decrease in the expression of all three genes was observed and, in the case of salt stress, a significant decrease in *Bv*AAO_10 expression was observed compared to the control and salt shock-treated samples up to the 7th day of the experiment. A different situation was observed in *Beta vulgaris* cv. Huzar. Here, increases and decreases in expression were observed alternately and their directions were mostly opposite for the salt stress treated and non-salt stress treatments. Taking into account the fact that the comparison concerned the halophyte and the beet cultivar, it can be assumed that the important element of higher salinity tolerance is the suppression of the expression of ascorbate oxidase genes during the stress. It is also noticeable that the expression profile of the tested genes in the salt shock-treated samples is similar to that in the control, suggesting that the salt shock treatment of plants does not initiate changes in the regulation of transcription of AAO genes in most cases. Analyzing the impact of drought on the expression level of *Bv*AAO_3, *Bv*AAO_4 and *Bv*AAO_10 genes, we observe its decrease in the treated samples or no effect. The *Bv*AAO_3 gene in both beet species is characterized by a decrease in expression compared to the control, similar to the *Bv*AAO_4 gene in *Beta vulgaris* cv. Huzar. We can see that, in contrast to salinity, a cultivated beet genotype downregulates the expression of two AAO genes during drought, while *Beta maritima* only one. The sole literature data on the regulation of the expression level under salt and drought stress were presented by Batth et al. [49]. The researchers also showed different expression profiles depending on the AAO gene and the type of stress. Similar observations were made in the case of *Oryza sativa*. Here, the downregulation of the *Os*AAO2 gene under salt stress and the downregulation of the *Os*AAO2, *Os*AAO3 and *Os*AAO5 genes under drought conditions were observed. Conversely, in the case of most AAO genes in *Arabidopsis thaliana* and *Zea Mays*, salt stress and drought caused their over-expression [49]. These observations may also be due to differences in salt and drought stress tolerance by these three plant species, which result in different regulations of AAO gene expression.

Analyzing the effect of salinity on the level of methylation of the CpG islands of the promoter regions of the *Bv*AAO_3, *Bv*AAO_4 and *Bv*AAO_10 genes, we also observe differences depending on the intensity and type of stress, as well as the beet genotype. In the case of salinity, the methylation level of the CpG islands of the studied genes in *Beta vulgaris* cv. Huzar was much higher than in the halophytic ancestor. This observation does not translate into a decrease in the expression level of AAO genes in this genotype. The explanation for this may be the involvement of other epigenetic factors, which may directly or indirectly affect gene expression and/or modify the effects of DNA methylation [42,56,57]. Taking into account the salt stress, in all cases there was an increase in the level of CpG island methylation with the duration of the experiment. When analyzing the treatment of plants with drought, a higher degree of methylation of the CpG islands of the AAO genes is also observed in the beet cultivar. In *Beta martima*, on the other hand, these values are significantly higher than the levels of methylation in the salinity treatment. As there is no literature data on the regulation of AAO gene methylation levels in beet and other plant species, we cannot compare the obtained results. However, there is no doubt that, during salinity or drought, selected epigenetic elements are regulated to increase the level of DNA methylation and thus silence the expression of the ascorbate oxidase genes of two beet species.

Ascorbate is a very important component of plant cells due to its numerous functions. One of them is ROS detoxification, which is especially important under oxidative stress-generating conditions. ASC scavenges singlet oxygen, superoxide anion and hydroxyl radical. Moreover, it is electron donor in many necessary redox reactions [10]. Studies on transgenic plants with reduced ascorbate production indicate a significant increase in the amount of hydrogen peroxide, which exerts a destructive effect on many areas of cellular functioning [58]. In turn, it has been confirmed that exogenous application of ASC mitigates the effects of salinity and stimulates plant regeneration after stress, [59,60]. In our study, we observed an initial decrease in ASC concentration in leaves of *Beta maritima* treated with salinity and in leaves of both drought-treated beet genotypes, compared to the control. After this time, in these experimental systems, a significant increase in ASC concentration occurred, compared to the control. In turn, in leaves of *Beta vulgaris* cv. Huzar treated with salinity, an increase in ASC was observed, lasting from the 5th day of the experiment. The initial decrease in ASC could indicate that the pool of this type of antioxidant was partially used up, as a result of the ROS detoxification. Similar results were observed, by Chen et al.， 2021 [61]. The increase in total ASC concentration over time may indicate that mechanisms leading to an increase in its amount under stress conditions are activated. One such mechanism may be the reduction in the expression level of genes encoding AAO, which results in a decrease in the amount and activity of this enzyme. The increase in ASC concentration under abiotic stress conditions was also confirmed in other studies [62,63]. Analyses aimed at the determination of AAO activity in our experimental system showed a decrease in the activity of this enzyme over time of treatments with stress-inducing factors. This correlated with our results, demonstrating the reduction of AAO gene expression under stress conditions. AAO regulates the redox state of the apoplast [10]. It has been proven that reducing the activity of this enzyme is promotes increase in plant resistance to oxidative stress since, under limited AAO activity, larger amounts of reduced ascorbate is maintained and this may be used for ROS scavenging [4,51]. Consequently, increasing the ascorbic acid concentration allows for a reduction in the amount of H_2_O_2_ and, consequently, reduces sensitivity to stress [10,64]. It should be kept in mind that the response to salinity differs between the two beet genotypes analyzed. *Beta maritima* is a halophytic plant and thus is more resistant to salinity than *Beta vulgaris* cv. Huzar. Therefore, it is possible to observe the activation of other mechanisms of the response to salinity, which involve different rate of ascorbate production and/or consumption.

## 4. Materials and Methods

### 4.1. Identification of Ascorbate Oxidase Sequences in Beta vulgaris

The source of the gene sequences analyzed in the study of *Beta vulgaris subsp. vulgaris* was the reference genome of this plant with the number RefBeet-1.2.2 deposited at GenBank (https://www.ncbi.nlm.nih.gov/gene/?term=ascorbate%20oxidase%20beta%20vulgaris; accessed on 5 January 2021).

### 4.2. Bioinformatic Analysis

The open reading frames (ORFs) of AAO genes were predicted using the ORF finder (https://www.ncbi.nlm.nih.gov/orffinder/; accessed on 10 January 2021). For a few, the longest ORF was chosen. Parameters such as molecular weight (MW), amino acid (AA) composition and theoretical isoelectric point (pI) were determined using the ProtParam of the Expasy package (https://web.expasy.org/protparam/; accessed on 21 February 2021). The presence of signal peptide was predicted with SignalP-5.0 (http://www.cbs.dtu.dk/services/SignalP/; accessed on 21 February 2021) and subcellular targeting was predicted with Plant mPLoc (http://www.csbio.sjtu.edu.cn/bioinf/plant-multi/; accessed on 21 February 2021) and Yloc (https://bio.tools/yloc; accessed on 21 February 2021). The chromosomal positions and structure of the beet AAO genes were acquired from the NCBI genome browser. Selected cis-regulatory elements were identified, in the sequences, using the Database of Plant Cis-acting Regulatory DNA Elements—New PLACE (https://www.dna.affrc.go.jp/PLACE/?action=newplace; accessed on 30 February 2021), while the potential sites recognized by selected salt-related transcription factors were determined using the Plant Promoter Analysis Navigator PlantPAN 2.0 program (http://plantpan2.itps.ncku.edu.tw/; accessed on 30 February 2021).

### 4.3. Sequence Alignment and Phylogenetic Analysis

Alignment and phylogenetic trees were constructed in CLC Sequence Viewer 8.0 (Qiagen, CLC bio A/S, Aarhus, Denmark). Phylogenetic and molecular evolutionary analyses were conducted using the neighbor-joining method according to the amino acids sequence with 1000 bootstrap replicates.

### 4.4. Plant Material

*Beta vulgaris* ssp. *maritima (B. maritima)* and sugar beet, *Beta vulgaris* ssp. *vulgaris* cv. Huzar were used as plant material. *B. maritima* seeds were obtained from National Germplasm Resources Laboratory (Beltsville, MD, USA) and the sugar beet seeds from Greater Poland Sugar Beet Breeding–WHBC (Poznań, Poland). The seeds (5 per pot) were sown into pots filled with sand and vermiculite (1/1 *v*/*v*) and plants were watered regularly with half-strength Hoagland solution. Plants were cultured for four weeks in a growth chamber with a photoperiod of 16 h of light and 8 h of darkness with standard irradiation of 30 ± 5 µmol m^−2^ s^−1^, provided by T8 15 W 6500 K “Daylight” tubes (POLAMP, Ełk, Poland). The temperature regime was 25 °C during the day and 18 °C at the night. Subsequently, treatments with salinity or drought were performed.

### 4.5. Exposing Plants to Salinity

Salt treatment was started when the first pair of mature leaves were fully developed. Salt shock—Plants were watered, over an 11-day-long treatment period, in two-day-long intervals, with half-strength Hoagland medium supplemented with 150 mM NaCl. Untreated controls were watered with a NaCl-unsupplemented medium. The leaf samples representing intact leaf blades were collected for analysis on first, third, fifth, seventh, ninth and eleventh day of the experiment (Skorupa et al., 2019). Salt stress—Over the first 11 days of treatment, plants were watered in two-day-long intervals, with half-strength Hoagland medium supplemented with increasing concentrations of NaCl, until the final concentration of 150 mM NaCl was reached. (first day of treatment–25 mM NaCl, third day–50 mM NaCl, fifth day–75 mM NaCl, seventh day–100 mM NaCl, ninth day–125 mM NaCl, eleventh day–150 mM NaCl). Untreated controls were watered with a NaCl-unsupplemented medium. Plants were watered with 200 mL of solution per 2 l of sand/vermiculite mixture. Leaves representing the second pair of true leaves were collected for analysis. 

### 4.6. Exposing Plants to Drought

Drought treatment was started when the first pair of mature leaves were fully developed. Drought shock—Over 11 days of treatments, plants were not watered. Untreated controls were watered with a 30 mL half-strength Hoagland medium. The leaf samples representing intact leaf blades were collected for analysis on the first, third, fifth, seventh, ninth and eleventh days of the experiment. Drought stress—Over 11 days of treatment, plants were watered in two-day-long intervals, with half-strength Hoagland medium with decreasing water volume. (1st day of treatment–30 mL/per pot, third day–25 mL/per pot, fifth day–20 mL/per pot, seventh day–15 mL/per pot, ninth day–10 mL/, eleventh day–5 mL/per pot). Untreated controls were watered with a 30 mL NaCl-unsupplemented medium. Plants were watered with 200 mL of solution per 2 l of sand/vermiculite mixture. Leaves representing the second pair of true leaves were collected for analysis. 

62 experimental variants were analyzed in the experiments. Two variants were represented by *Beta maritima* and *Beta vulgaris* cv. Huzar leaves on the day of starting the experiment, before applying the stresses. Sixty experimental variants were represented by the leaves of two beet species grown under control conditions or treated with drought or salinity on the respective leaf harvest days. In the case of RT qPCR analyses and methylation analyses, the number of analysis samples was: 62 experimental variants × 3 biological replicates × 3 technical repetitions = 558. For the determination of total ascorbate and AAO activity, the number of analyzed samples was: 62 experimental variants × 6 biological replicates × 3 repetitions technical = 1116.

### 4.7. RT qPCR Analysis

Total RNA was isolated from the same leaf samples using the TriPure reagent (Roche Diagnostics GmbH, Mannheim, Germany). Total RNA preparations were subjected to DNase treatment, which was carried out using DNase I (Thermo Scientific, Vilnius, Lithuania) according to the manufacturer’s instructions. The first-strand cDNA was synthesized from 1 μg of total RNA using random hexamers and Transcriptor High Fidelity cDNA Synthesis Kit (Roche Diagnostics GmbH, Mannheim, Germany), following the manufacturer’s instructions. Gene-specific primers were designed using Primer3Plus software. To ensure the correctness of the quantification, the expression was normalized to the β-actin reference gene. To determine the PCR efficiencies, standard curves for both target and control genes were obtained using a series of cDNA dilutions as a template. RT qPCR was performed in three biological replicates, each of which consisted of three technical replicates, using FastStart SYBR Green Master Mix, following the standard PCR program suggested by the manufacturer (Roche Diagnostics GmbH, Mannheim, Germany). The relative level of gene expression was calculated according to the Pfaffl method. The control against which the calculations were performed was represented by the leaves of the two beet genotypes at the start of the experiment, before the start of the stress treatment. Blank controls without a cDNA template were included in every experimental setup. A list of PCR primers used for experimental is provided in Table S1. The ordinal number of the primer sequences in Table S1 for the *Bv*AAO_3, *Bv*AAO_4, *Bv*AAO_10 0 genes, respectively, are 3, 4 and 10. The qPCR efficiency values for the genes analyzed are summarized in Table S2.

### 4.8. Analysis of the CpG Island Methylation Level of Selected Beet AAO Genes

The level of methylation of the CpG islands of the promoter regions of the *Bv*AAO_3, *Bv*AAO_4 and *Bv*AAO_10 genes was analyzed. Measurements were taken on the 3rd, 7th and 11th day of treating plants with saline or drought. The location of the islands and the analysis of their methylation level was determined according to Skorupa et al. 2021 [24]. The primer sequences for analysis are listed in the Table S1. The qPCR efficiency values for the genes analyzed are summarized in Table S2.

### 4.9. Determination of the Concentration of Total Ascorbate

150 mg of leaf tissue was extracted in a mortar with 1 mL of 5% TCA. The samples were then centrifuged at 14,000× *g* and 4 °C for 10 min. 16.8 μL of 10 mM DTT and 80 mM K_2_HPO_4_ were added to 135 μL of supernatant and incubated for 5 min at RT. In the next step, 40 µL of 85% H3PO4, 685 µL of 0.5% α, α′-dipyridyl and 140 µL of 1% FeCl_3_ were added successively, followed by incubation for 30 min at RT and centrifugation at 14,000× *g* for 5 min. The absorbance values were measured spectrophotometrically at a wavelength of 525 nm (Analytik Jena, Jena, Germany). The blank was a sample containing water with 5% TCA, instead of a plant extract. Ascorbate concentrations (μg/mL) were read from a standard curve prepared from a series of ascorbic acid concentrations. Leaf ascorbate concentrations are expressed as µg ascorbate/gFW.

### 4.10. Measurement of the Enzymatic Activity of Ascorbate Oxidase

200 mg of plant tissue was homogenized in 1 mL of extraction buffer (66 mM Na^+^/K^+^ phosphate buffer pH 6.1, 0.5 mM EDTA) and centrifuged for 10 min at 10,000× *g* at 4 °C. The AAO activity was calculated on the basis of the change in absorbance at the wavelength of 265 nm for 60 s, in the reaction mixture with the following composition: 960 μL of the buffer (66 mM Na^+^/K^+^ phosphate buffer pH 6.1), 20 μL of the sample and 20 μL of 2.5 mM acid ascorbic acid. The calculations were made using the millimolar absorption coefficient for ascorbic acid at 265 nm, which is 14 mM^−1^ cm^−1^. The enzyme activity was expressed in AAO, nmol ASC mg of total protein^−1^ min^−1^].

### 4.11. Statistics

Statistical significance of differences between mean values were determined with two-way ANOVA followed by Tukey’s HSD test in SigmaPlot 2008 (Systat Software, Palo Alto, Santa Clara, CA, USA). Differences at the level of *p* < 0.01 were considered as significant. Mean and standard deviation were calculated. Error bars shown in all figures represent the standard deviation calculated from all repetitions of each experiment.

## 5. Conclusions

The response of a sugar beet cultivar *Beta vulgaris* cv. Huzar and its halophytic ancestor *Beta maritima* to drought and salinity is manifested by downregulating the expression of ascorbate oxidase genes. This correlated with a decrease in AAO enzyme activity in a given experimental setup. Silencing of the AAO gene expression is most pronounced in the downregulation of AAO transcripts in *Beta maritima* under salinity, compared to a crop and conversely, under drought, in *Beta vulgaris* cv. Huzar. Considering the high level of drought and salinity stress tolerance in wild beet, *Beta maritima* and relatively moderate tolerance to these stresses in a beet crop variety, suppression of AAO gene expression in beet leaves under stress conditions may contribute to the mechanism of increasing salinity and drought tolerance. Moreover, it can be concluded that the downregulation of *Bv*AAO_10 expression plays a key role in response to salinity treatment in *Beta maritima*, whereas downregulation of the *Bv*AAO_3 gene is predominant under drought in both beet genotypes. This phenomenon may be involved in determining the high tolerance of beet to salinity and drought.

## Figures and Tables

**Table 1:** Characteristics and properties of AAO genes and the proteins they encode in *Beta vulgaris*.

	Name in This Work	Name/ GeneID	Location	ORF	Exons	AA	MW (kDa)	pI	Signal P	Plant- mPLoc	Yloc
**1**	*Bv*AAO_1	LOC104890152	Chromosome 4, NC_025815.2 (3428816–3432297)	2253	4	557	62.94	8.98	Yes, 24–25 (0.96)	Cell membrane	Peroxisome (62.51%)
**2**	*Bv*AAO_2	LOC104890153	Chromosome 4, NC_025815.2 (3436425–3439102)	2145	4	558	63.30	8.79	Yes, 25–26 (0.94)	Cell membrane	Cytoplasm (86%)
**3**	*Bv*AAO_3	LOC104892300	Chromosome 5, NC_025816.2 (3577518–3582274)	2478	7	549	61.00	8.40	Yes, 24–25 (0.93)	Cell membrane	Peroxisome (64.50%)
**4**	*Bv*AAO_4	LOC104895593	Chromosome 6, NC_025817.2 (8750906–8755896)								
		variant X1		2063	9	538	59.68	9.41	Yes, 24–25 (0.96)	Cell membrane	Cytoplasm (74.22%)
		variant X2		2053	9	538	59.68	9.41	Yes, 24–25 (0.96)	Cell membrane	Cytoplasm (74.22%)
**5**	*Bv*AAO_5	LOC104895712	Chromosome 6, NC_025817.2 (10362403–10365259)	2095	5	588	65.90	6.68	Yes, 35–36 (0.57)	Cell membrane	Cytoplasm (85.23%)
**6**	*Bv*AAO_6	LOC104895596	Chromosome 6, NC_025817.2 (8771987–8776770)	2044	8	537	60.78	6.71	Yes, 24–25 (0.78)	Cell membrane	Cytoplasm (87.40%)
**7**	*Bv*AAO_7	LOC104895595	Chromosome 6, NC_025817.2 (8760298–8765330)								
		variant X1		2054	8	542	61.25	9.06	Yes, 24–25 (0.93)	Cell membrane	Extracellular space (98.22%)
		variant X2		1798	8	542	61.25	9.06	Yes, 24–25 (0.93)	Cell membrane	Extracellular space (98.22%)
		variant X3		1884	8	542	61.25	9.06	Yes, 24–25 (0.93)	Cell membrane	Extracellular space (98.22%)
**8**	*Bv*AAO_8	LOC104900974	Chromosome 8, NC_025819.2 (8345583–8354836)								
		variant X1		1989	8	548	62.61	9.84	Yes, 29–30 (0.92)	Cell membrane	Cytoplasm (75.26%)
		variant X2		2024	8	543	62.29	9.76	Yes, 24–25 (0.91)	Cell membrane	Cytoplasm (80.90%)
**9**	*Bv*AAO_9	LOC104903081	Chromosome 9, NC_025820.2 (11718104–11729554)	2063	5	588	65.60	8.55	No	Cell membrane	Cytoplasm (91.22%)
**10**	*Bv*AAO_10	LOC104903859	Chromosome 9, NC_025820.2 (32099171–32106493)	2117	8	537	60.84	8.96	Yes, 22–23 (0.97)	Cell membrane	Cytoplasm (64.34%)
**11**	*Bv*AAO_11	LOC104884409	NW_017567596.1 (164256–159396)	2176	7	539	59.82	9.40	Yes, 20–21 (0.95)	Cell membrane	Extracellular space (82.94%)
**12**	*Bv*AAO_12	LOC104884407	NW_017567596.1 (158898–154368)	3014	7	539	59.89	9.46	Yes, 20–21 (0.93)	Cell membrane	Secretory Pathway (96.60%)

## Supplementary Materials

The following supporting information can be downloaded at: https://www.mdpi.com/article/10.3390/ijms232112773/s1.
