# Supplementary material for: Characteristic of the Ascorbate Oxidase Gene Family in Beta vulgaris and Analysis of the Role of AAO in Response to Salinity and Drought in Beet
Source: Int J Mol Sci. 2022 Oct 23;23(21):12773. doi: 10.3390/ijms232112773 (PMC9654295; doi:10.3390/ijms232112773)
Supplement: Supplementary file 1 [file ijms-23-12773-s001.zip › ijms-1929197-supplementary.pdf]

Characteristic of the Ascorbate Oxidase Gene Family in *Beta vulgaris* and Analysis of the Role of AAO in Response to Salinity and Drought in Beet.

Monika Skorupa, Joanna Szczepanek, Seher Yolcu, Justyna Mazur, Andrzej Tretyn, Jarosław Tyburski

Table S1 . Primer sequences and the lengths of PCR products.

	gene symbol	primers sequence 5′->3′		length of the PCR product [bp]
1	*Bv*AAO_1	F	CTTTTCGCTTGGCAGGATAC	174
		R	TCCACATCCCTACGTTGTCA	
2	*Bv*AAO_2	F	ACCACTACGGGCAGATCAAC	126
		R	GCTTCAATGGGGTATCAACG	
**3**	***Bv*AAO_3**	**F**	**TAGCCGACCCTTGATACCTG**	**136**
		**R**	**ATCAGGGGAAGGCAAATCTT**	
**4**	***Bv*AAO_4**	**F**	**GGCTGCTTCTCACCATGATT**	**131**
		**R**	**GACTGGCTTGGGTCCATTTA**	
5	*Bv*AAO_5	F	TCAAAGACCGCCTCCTAATG	175
		R	CGGGTGAGTATCGCTGGTAT	
6	*Bv*AAO_6	F	TGATATGCCTACGCCTGATG	171
		R	GAACCTTCCACCTCAACCAA	
7	*Bv*AAO_7	F	ATGGTGTTTGGCAGAGGAAG	144
		R	GCATCAATGTGGAAGGGAAG	
8	*Bv*AAO_8	F	CGTCACACTCGCCCTATGTA	127
		R	GCCTGGGAAGTAAAGCATGA	
9	*Bv*AAO_9	F	ACCCGAAAACTTCCCTTCAT	143
		R	CATTAGCACCCAAGGCATTT	
**10**	***Bv*AAO_10**	**F**	**CACAGCGGATCAAGAAGTCA**	**103**
		**R**	**GTTGGAGCCCTGATAGTGGA**	
11	*Bv*AAO_11	F	TGCGGACATTGTTGTGTTTT	139
		R	AAAATGCCCTGTTGACGAAC	
12	*Bv*AAO_12	F	GTTTTCAGTGTGGGGAGCAT	148
		R	AACCATCCAAATGCCAACTC	
13	β-actin	F	ATCCAGGCCGTTCTTTCTCT	144
		R	ACGACCAGCAAGATCCAAAC	
14	CpG island of *Bv*AAO_3	F	TGCATATGAGCTGGACCAAC	244
		R	TCATGGTCTTCATGGCTCCT	
15	CpG island of *Bv*AAO_4	F	TGTTTTTGGCTTTGGGACGG	307
		R	GTCAACCTTTTCGCGGTCTT	
16	CpG island of *Bv*AAO_10	F	TCATAACCCTAGACATGGCC	177
		R	CGGTTCATGTTTGGCTCACT	

Table S2. The qPCR efficiency values for the genes analyzed.

Gene/Region	Efficiency
***Bv*AAO_3**	1,89
***Bv*AAO_4**	1,944
***Bv*AAO_10**	1,916
β-actin	1,946
CpG island of *Bv*AAO_3	1,966
CpG island of *Bv*AAO_4	1,920
CpG island of *Bv*AAO_10	1,933
